# Minimally invasive esophagectomy: clinical evidence and surgical techniques

**DOI:** 10.1007/s00423-020-02003-w

**Published:** 2020-10-07

**Authors:** C. Mann, F. Berlth, E. Hadzijusufovic, H. Lang, P. P. Grimminger

**Affiliations:** Klinik für Allgemein-, Viszeral und Transplantationschirurgie, Johannes Gutenberg-Universität Mainz, Langenbeckstr. 1, 55131 Mainz, Germany

**Keywords:** Esophagectomy, Minimal invasive esophagectomy, Esophageal cancer, Robot-assisted minimal invasive esophagectomy

## Abstract

**Background:**

Surgical esophagectomy plays a crucial role in the curative and palliative treatment of esophageal cancer. Thereby, minimally invasive esophagectomy (MIE) is increasingly applied all over the world. Combining minimal invasiveness with improved possibilities for meticulous dissection, robot-assisted minimal invasive esophagectomy (RAMIE) has been implemented in many centers.

**Purpose:**

This review focuses on the development of MIE as well as RAMIE and their value based on evidence in current literature.

**Conclusion:**

Although MIE and RAMIE are highly complex procedures, they can be performed safely with improved postoperative outcome and equal oncological results compared with open esophagectomy (OE). RAMIE offers additional advantages regarding surgical dissection, lymphadenectomy, and extended indications for advanced tumors.

## Introduction

As 6th most fatal malignancy with approximately 500,000 new cases worldwide per year, esophageal cancer represents a serious oncological burden [1, 2]. Nowadays, multimodal therapeutic approaches—with surgery as cornerstone—achieve 5-year-survival rates up to 50% [3, 4]. Due to the high technical complexity of the totally minimally invasive esophagectomy (MIE), open esophagectomy or hybrid esophagectomy (laparoscopic and open thoracic) is still common practice for resectable esophageal cancer. Thereby, two factors appear extraordinarily challenging when performing an esophagectomy. Firstly, the esophagus and stomach are embedded in both the thorax and the abdomen. While performing an oncological esophagectomy, both abdominal and thoracic lymph node compartments must be dissected for a radical two-field lymphadenectomy. Therefore, a two-compartment intervention is inevitable. Secondly, the thoracic esophagus is located right next to delicate and essential structures like the trachea and the bronchi, the cardiac atrium, and large vessels like the aorta, azygos vein, and pulmonary vein as well as crucial nerve structures. Hence, in regard to a recent international benchmark study, surgical esophagectomy—although performed in high-volume centers—is accompanied by an overall complication rate up to 60% [5]. The procedure needs to be performed in a way allowing most precise and exact preparation while keeping it as little invasive as possible to avoid complications, without compromising oncological principles. These requirements are leading towards minimally invasive surgery. This article reviews the origin and current clinical evidence of MIE as well as the surgical techniques and limitations.

## Minimally invasive esophagectomy—development and present clinical evidence

Facing high postoperative morbidity and mortality in open surgery, minimally invasive esophagectomy was firstly introduced with a series of five cases in 1992 [6]. Cuschieri et al. presented their technique using camera-assisted thoracoscopic access combined with laparotomy. Collard et al. reported in 1993 a series of 9 patients undergoing a thoracoscopic approach [7]. This technique had been adopted by more surgeons worldwide creating the category of “hybrid techniques” referring to the combination of laparotomy and thoracoscopy or laparoscopy and thoracotomy [8, 9]. A randomized controlled trial was conducted to compare the hybrid approach (laparoscopic abdominal phase, open thoracic phase) to open esophagectomy: the MIRO trial. Its long-term results—showing equal results for both arms—were published in 2019 [10]. Primary endpoint of the MIRO trial was the frequency of perioperative complications of grade II or higher according to the modified Clavien-Dindo classification (MCDC) [11]. Results showed a significantly lower rate of major complications (36%), especially pulmonary complications (18%), in the hybrid group compared with the open transthoracic esophagectomy group (64% and 30%, respectively) [9, 12]. However, many concerns have been raised over these outcomes since the open thoracic part, which is to some extent responsible for the high rates of pulmonary complications, remains equal to the open approach.

While randomized controlled trials stepwise include the use of minimally invasive techniques in protocols by hybrid procedures, other surgeons would have developed MIE procedures in order to maximally deliver the benefit of minimal invasiveness.

A cornerstone towards MIE was the laparoscopic transhiatal esophagectomy approach without thoracotomy and creation of the anastomosis in the neck as reported by DePaula et al. in 1995 and Luketich et al. in 1998 [13, 14]. The transhiatal approach seemed to have advantages according to postoperative morbidity, but later showed inferior long-term survival rates compared with transthoracic esophagectomy and was therefore no longer performed as standard procedure in oncological esophagectomy [15].

For that reason, MIE as totally minimally invasive transthoracic esophagectomy was developed. After presenting successful MIE cases in 1998 by Luketich et al. [16], the first large series of totally minimally invasive patients was published in 2002 [17]. In this single-center experience, a remarkable low mortality rate (1.4%) and short hospital stay (7 days) compared with most open series were observed.

After a period of proofing the feasibility and safety of MIE, the multi-center randomized TIME trial was set up to compare total MIE with the gold standard by that time, open esophagectomy [18]. Starting in 2012, the TIME trial recruited 115 patients from seven centers in four nations. MIE was performed beginning with laparoscopic gastric mobilization and abdominal lymphadenectomy followed by thoracoscopic dissection of the esophagus in prone position. Anastomosis was then performed in the neck (McKeown procedure). This study assessed several perioperative outcomes as well as oncological outcome and quality of life. It revealed a superiority of MIE compared with open esophagectomy regarding intraoperative blood loss, acute immunological response, postoperative pulmonary infections, length of hospital stay, postoperative pain scores, and quality of life. The primary endpoint pneumonia by contrast was questioned due to missing standardization and the relatively high rate of pneumonia in the open esophagectomy group (36%). Additionally, lymph node yield and long-term oncological outcomes in terms of disease free and overall survival have proven to be equivalent compared with open esophagectomy [19, 20].

Following the TIME-trial, which included a relatively limited number of patients (59 vs. 56 cases), several systemic meta-analyses have compared perioperative and long-term outcomes between MIE and OE [21–28]. All of them mostly reflect the findings of TIME trial. Six out of the 8 meta-analyses reported significantly lower intraoperative blood loss in MIE, the amount of total complications was lower in four analyses as well as the duration of postoperative hospital stay. Specifically, pulmonary complications were found to be significantly lower in four meta-analyses. TIME-trial reported 36% of patients in the open esophagectomy group having pulmonary infection in-hospital compared with 12% in the minimally invasive group (relative risk [RR] 0.35, 0.16–0.78; *p* = 0.005) [20]. In 2016, Yibulayin et al. analyzed fifty-seven studies containing 15,790 cases of resectable esophageal cancer, the highest numbered meta-analysis. It found a reduction of overall postoperative complications in patients treated with MIE (41.5%) vs. OE (48.2%). Specifically, pulmonary complications (OR = 0.527, 95% CI = 0431~0.645, *p* < 0.05), cardiovascular complications (OR = 0.770, 95% CI = 0.681~0.872, *p* < 0.05), and surgical technology-related (STR) complications (OR = 0.639, 95% CI = 0.522~0.781, *p* < 0.05), as well as in-hospital mortality (OR = 0.668, 95% CI = 0.539~0.827, *p* < 0.05), were found to be lower in the MIE group. In accordance to TIME trial, the number of harvested lymph nodes did not show significant differences.

Regarding total operation time, performing MIE seems to take significantly longer, most probably due to the surgeons’ learning curve to reach full technical proficiency [29]. Xiong et al. reported an average operation time of 334.5 min performing MIE vs. 292.5 min performing OE. Forty-six studies (6260 cases) of the analysis from Yibulayin et al. had higher operative time in the MIE group (*p* < 0.05). It can also be assumed that the lower blood loss during resection is surgically traded with a longer operation time. Even minor bleedings can compromise the magnified view in MIE; therefore, surgeons tend to work on hemostasis very thoroughly. Accordingly, a longer operation time and lesser blood loss are often observed compared with open approach.

Additionally, the reintervention rate after MIE was found to be significantly higher in some studies analyzing short-term outcomes [30–33]. Silhag et al. found 9.9% cases requiring postoperative reintervention in the MIE group vs. 4.4% cases in the OE group (*p* < 0.001) [30]. This rate might also be linked to the abovementioned learning curve. Van Workum et al. investigated the morbidity that is associated with the learning curve of MIE in 2019 using the anastomotic leakage rate of intrathoracic-created anastomoses as primary outcome [29]. Starting with an anastomotic leakage rate of 18.8%, it took an average of 119 cases to reach the plateau for an anastomotic leakage rate of about 8%.

Finally, reduced mortality after MIE could not be shown by most analyses and studies. Only Yibulayin et al. reported strong evidence for a lower 30-day mortality. Long-term outcomes have shown to be at least equal to open esophagectomy. Dantoc et al. describe a higher 1-year survival rate and an equal 3-year survival rate. Osugi et al. and Smithers et al. compared 3-year survival rates after a 3-field lymphadenectomy, respectively, a 2-field lymphadenectomy with equal survival rates in both groups. Guo et al. found an even better 2-year survival rate after minimal invasive surgery.

To finally investigate if and how patients benefit from a totally minimally invasive approach, another trial was set up only in the UK: the ROMIO study (Randomised Oesophagectomy: Minimally Invasive or Open) [34, 35]. This study compares open to hybrid with minimally invasive esophagectomy and the pilot trial recruited 104 patients in the three arms in a period of 21 months. As all three approaches are included in this trial, it is also aimed to show the potential additional benefit from MIE over hybrid procedure.

## RAMIE—development and clinical evidence of robot assisted esophagectomy

Technically, challenging aspects of MIE procedure are rigid instruments in a relatively inflexible chest cavity, limited access to the remote surgical field, and limited range of view. To overcome these limitations, robotic-assisted surgical systems were established and found their way into esophageal surgery in the early 2000s. In 2006, first case series were published introducing RAMIE, especially the thoracoscopic phase, as a safe and feasible procedure [36, 37]. However, proof was only based on retrospective and prospective case series [38]. To evaluate the value of robotic-assisted intervention in esophageal surgery, the ROBOT trial (robotic-assisted thoracoscopic procedure with open abdomen procedure (robotic hybrid esophagecotmy) vs. open esophagectomy; both with neck anastomoses) was initiated in 2012 [39]. As a one-center randomized controlled trial with 112 patients, it compared RAMIE and OE taking the occurrence rate of overall complications (according to the modified Clavien-Dindo classification grade 2–5 [11]) as primary outcome. RAMIE procedure was accompanied by a significantly lower overall complication rate (59 vs. 80%), most likely resulting from the reduction of pulmonary (32 vs. 58%) and cardiac (47 vs. 22%) complications [40]. In addition to that, postoperative pain, short-term quality of life, and short-term postoperative functional recovery were significantly better in RAMIE group [41]. In terms of surgical radicality, lymph node yield and overall survival rates were found to be equal. Thus, RAMIE offers several short-term profits while maintaining high oncological standards compared with open esophagectomy. Struggling with the same points of criticism as the MIE procedure—long operation time and long learning curve—it should be at least seen as an appropriate alternative to MIE.

Finally, the cost aspect of this new technology in the European health care system needs to be mentioned. The robot platform not only is a high investment at first but also comes along with high maintenance costs. First experiences show that although costs per procedure are higher, total costs (including the treatment of complications) of the RAMIE patients may be lower, since complications are reduced. Additionally, technical progress with possible usage of artificial intelligence and developments beyond our current imaginations should not be underestimated.

In the end, clear evidence for superiority of RAMIE over MIE by prospective controlled studies are not yet available, but there are already studies in progress or initiated (ROBOT-2 trial (NCT04306458) and, e.g., the REVATE study). Since the amount of significantly measurable improvements by robotic assistance compared with conventional MIE is expected to be rather small, high numbers of patients need to be treated for a trial to compare both procedures. The results of the abovementioned trials (e.g., the REVATE study and ROBOT-2 trial [42]) which compare RAMIE with conventional MIE are eagerly awaited [43, 44].

## Surgical aspects—anastomosis, lymph node yield, and extended indications

Still to be discussed are the key elements of esophagectomy: anastomotic technique and lymph node yield. We point out differences and possibilities offered by MIE and RAMIE.

### Anastomotic techniques and leakage in MIE and RAMIE

The most threatening and lethal significant complication after esophageal resection is the anastomotic leakage. For fair comparison of occurrence and severity of anastomotic leakage following esophagectomy, two principally different techniques must be discriminated. Neck anastomosis can be performed with or without cervical lymphadenectomy and includes resection of the whole intrathoracic esophagus. This anastomosis is routinely performed through left cervical incision and does not differ in open or minimally invasive approach (Mc Keown procedure). The high intrathoracic anastomosis, in contrast, is performed during the thoracic phase of esophagectomy above the azygos vein level and it is performed intracorporeally during MIE (Ivor Lewis procedure) [45–47]. Therefore, if applied, it appears as an extra technical challenge in MIE. It is discussed, if anastomotic leakage after high intrathoracic esophagectomy is associated with higher severity of complication and whether neck anastomosis is associated with a higher rate of leakages [48].

In the abovementioned meta-analyses, comparing MIE with OE five meta-analyses including Yibulayin et al. showed no difference in occurrence rate of postoperative anastomosis leakage rate. Only Nagpal et al. (2010) [28] reported a lower rate of anastomotic leakages. However, they only included 1284 cases. Nevertheless, none of these meta-analyses discriminates between intrathoracic and cervical anastomosis—studies analyzing both types were equally included. When performed in the neck, all anastomoses are created using an open neck approach regardless of the prior approach. One could only argue about lower anastomotic leakages after MIE on account of smaller operational trauma. Debatable remains the minimally invasive created intrathoracic anastomosis. Due to greater technical difficulties, one could expect higher rates of complications. The meta-analysis of Zhou et al. in 2015 [49] which analyzes only anastomotic leakages after MIE and OE distinguished between intrathoracic and cervical anastomoses. Both types of anastomotic procedure had a comparable rate of anastomotic leakage with no significant difference comparing MIC with open approach.

At the same time as surgical techniques developed towards minimally invasive procedures, endoscopic possibilities were similarly improved. Since a variety of treatment options are nowadays available, the re-operation rate as well as the mortality after leaks has been dramatically decreased [50].

Regarding a potential higher anastomotic leakage rate using robotic assistance in comparison with OE, van der Sluis et al. found no significant difference in their prospective randomized study in 2019 [40]. Still, in this study as well as in the ROBOT trial, all anastomoses were created in the neck and not with robotic assistance. The critical anastomosis, comparing anastomotic leakage rates after esophagectomy, remains the thoracic anastomosis, which seems to be more challenging to create during MIE. Thereby, the robotic system offers some unique advantages not reached by the conventional minimal invasive approach. Our own data of the first 100 robotic-assisted Ivor-Lewis esophagectomies with intrathoracic-created end-to-end circular-stapled anastomoses suggest leakage rates of 8% [51, 52]. These numbers hint at lower leakage rates compared with leakage rates around 24% of OE and Mc Keown procedures with neck anastomoses [53].

Owing to stiff instruments with less flexibility due to the osseous thorax and limited range of motion, the third possible anastomotic technique—hand-sewn intrathoracic anastomoses—is especially difficult to perform using conventional MIE. Therefore, the abovementioned circular-stapled anastomoses have become common practice—although some studies hint at higher rates of postoperative benign structure formation after circular-stapled anastomosis [54–57]. These benign structure formations cause long healing courses for the patient with the need for many endoscopic dilatation treatments and deteriorating nutritional status due to dysphagia. The robot translates the surgeons hand movements on the console to the surgical instruments with flexible joints making a larger range of motion possible. This is of great benefit and makes hand sewing easier to perform. Additionally, it allows safe hand-sewn reinforcement stitches at any part of the operation. Prospective randomized studies need to be conducted to compare hand sewing vs. stapling anastomosis regarding anastomotic leakages and benign structure formation in RAMIE.

### Lymphadenectomy in MIE and RAMIE

Regarding overall survival, the extent of lymphadenectomy during esophagectomy is crucial [58]. It is stated that a high lymph node yield (at least 15 lymph nodes) is regardless of the preoperatively staged nodal status, histopathological entity (adenocarcinoma or squamous cell carcinoma), and surgical approach, associated with improved overall survival [59]. A meta-analysis by Visser et al. confirmed these findings in western and eastern populations with and without neoadjuvant therapy [60]. RAMIE offers meticulous and safe dissection in delicate regions allowing sufficient extent of lymphadenectomy even in highly complex cases, e.g., after neoadjuvant treatment or definitive radiation. The group in our institution found an average of 28 lymph nodes resected using the Davinci Xi [52] and a trend towards improved lymphadenectomy in RAMIE compared with MIE [61]. In RAMIE, the possibility of near-infrared fluorescent imaging (NIR) is additionally helpful: either preoperatively injected labeled colloid or indocyanine green dye (ICG) in submucosa can visually support the identification of lymph nodes or vessel [62]. Modern robotic platforms standardly offer this modality. However, a matter of debate remains the necessity of lymphadenectomy in the paratracheal region, which many surgeons only perform for mid- and proximal tumors. When using robotic assistance, these regions can be reached easier without risking unnecessary iatrogenic damage.

Additionally, the robotic system offers an extension of indications for patients with locally advanced tumors. Tumors with a preoperative stage < cT4b are often treated with definitive or extended chemoradiation. After such treatment (e.g., radiation with 50 Gy compared with 41.5 Gy used in the CROSS regimen), the tissue in the field of interest shows fibrotic transformation hindering future surgical dissection. However, robotic assistance with better access to the upper mediastinum can help resecting advanced, highly pretreated and proximal tumors while avoiding damage to essential structures and respecting the oncological resection margins. Thus, patients with primary advanced local tumors and major response to chemoradiation could be offered a surgical approach with radical resection. In a first Dutch case series, nine of ten patients with T4b tumors, high mediastinal tumors, and lymph node metastases after neoadjuvant treatment were treated successfully with RAMIE [63].

## Conclusion

Until today, the multimodal concept treating esophageal cancer with a combination of chemo(radio)therapy and surgery is a most promising approach for extended long-term survival. Thereby, minimally invasive surgery proved to have reduced perioperative morbidity with equivalent oncological radicality and outcomes compared with open esophagectomy. There is a trend towards minimally invasive applied intrathoracic anastomoses; however, the benefit during the Ivor-Lewis procedure must be analyzed in further studies. Robotic assistance during esophageal surgery has been implemented as a safe and feasible procedure analog to conventional MIE. Prospective studies comparing RAMIE with MIE are still awaited. So far, it seems that in locally advanced tumors and complex cases, robotic assistance allows highest oncological radicality without risking more complications. Furthermore, today’s improvements of existing robotic platforms lay the foundation for future innovations such as artificial intelligence as well as data and skill sharing.

## References

[1] Bray F, Ferlay J, Soerjomataram I, Siegel RL, Torre LA, Jemal A (2018). Global cancer statistics 2018: GLOBOCAN estimates of incidence and mortality worldwide for 36 cancers in 185 countries. CA Cancer J Clin.

[2] Gupta B, Kumar N (2017). Worldwide incidence, mortality and time trends for cancer of the oesophagus. Eur J Cancer Prev.

[3] van Hagen P, Hulshof MC, van Lanschot J, Steyerberg EW, van Berge Henegouwen M, Wijnhoven BP, Richel DJ, Nieuwenhuijzen GA, Hospers GA, Bonenkamp JJ, Cuesta MA, Blaisse RJ, Busch OR, ten Kate F, Creemers GJ, Punt CJ, Plukker JT, Verheul HM, Spillenaar Bilgen EJ, van Dekken H, van der Sangen M, Rozema T, Biermann K, Beukema JC, Piet AH, van Rij C, Reinders JG, Tilanus HW, van der Gaast A, CROSS Group (2012). Preoperative chemoradiotherapy for esophageal or junctional cancer. N Engl J Med.

[4] Alderson D, Cunningham D, Nankivell M, Blazeby JM, Griffin SM, Crellin A, Grabsch HI, Langer R, Pritchard S, Okines A, Krysztopik R, Coxon F, Thompson J, Falk S, Robb C, Stenning S, Langley RE (2017). Neoadjuvant cisplatin and fluorouracil versus epirubicin, cisplatin, and capecitabine followed by resection in patients with oesophageal adenocarcinoma (UK MRC OE05): an open-label, randomised phase 3 trial. Lancet Oncol.

[5] Low DE, Kuppusamy MK, Alderson D, Cecconello I, Chang AC, Darling G, Davies A, D’Journo XB, Gisbertz SS, Griffin SM, Hardwick R, Hoelscher A, Hofstetter W, Jobe B, Kitagawa Y, Law S, Mariette C, Maynard N, Morse CR, Nafteux P, Pera M, Pramesh CS, Puig S, Reynolds JV, Schroeder W, Smithers M, Wijnhoven BPL (2019). Benchmarking Complications Associated with Esophagectomy. Ann Surg.

[6] Cuschieri A, Shimi S, Banting S (1992). Endoscopic oesophagectomy through a right thoracoscopic approach. J R Coll Surg Edinb.

[7] Collard JM, Lengele B, Otte JB, Kestens PJ (1993). En bloc and standard esophagectomies by thoracoscopy. Ann Thorac Surg.

[8] Davakis S (2020). Hybrid minimally-invasive esophagectomy for esophageal cancer: clinical and oncological outcomes. Anticancer Res.

[9] Mariette C, Markar SR, Dabakuyo-Yonli TS, Meunier B, Pezet D, Collet D, D'Journo XB, Brigand C, Perniceni T, Carrère N, Mabrut JY, Msika S, Peschaud F, Prudhomme M, Bonnetain F, Piessen G, Fédération de Recherche en Chirurgie (FRENCH) and French Eso-Gastric Tumors (FREGAT) Working Group (2019). Hybrid minimally invasive esophagectomy for esophageal cancer. N Engl J Med.

[10] Briez N, Piessen G, Bonnetain F, Brigand C, Carrere N, Collet D, Doddoli C, Flamein R, Mabrut JY, Meunier B, Msika S, Perniceni T, Peschaud F, Prudhomme M, Triboulet JP, Mariette C (2011). Open versus laparoscopically-assisted oesophagectomy for cancer: a multicentre randomised controlled phase III trial - the MIRO trial. BMC Cancer.

[11] Clavien PA, Barkun J, de Oliveira ML, Vauthey JN, Dindo D, Schulick RD, de Santibañes E, Pekolj J, Slankamenac K, Bassi C, Graf R, Vonlanthen R, Padbury R, Cameron JL, Makuuchi M (2009). The Clavien-Dindo classification of surgical complications: five-year experience. Ann Surg.

[12] Mariette C et al (2020) Health-related quality of life following hybrid minimally invasive versus open esophagectomy for patients with esophageal cancer, analysis of a multicenter, open-label, randomized phase III controlled trial: the MIRO trial. Ann Surg 271(6):1023–1029. 10.1097/SLA.0000000000003559 10.1097/SLA.0000000000003559 31404005

[13] DePaula AL (1995). Laparoscopic transhiatal esophagectomy with esophagogastroplasty. Surg Laparosc Endosc.

[14] Luketich JD, Nguyen NT, Schauer PR (1998). Laparoscopic transhiatal esophagectomy for Barrett’s esophagus with high grade dysplasia. Jsls.

[15] Hulscher JB (2002). Extended transthoracic resection compared with limited transhiatal resection for adenocarcinoma of the esophagus. N Engl J Med.

[16] Luketich JD, Nguyen NT, Weigel T, Ferson P, Keenan R, Schauer P (1998). Minimally invasive approach to esophagectomy. Jsls.

[17] Luketich JD (2003). Minimally invasive esophagectomy: outcomes in 222 patients. Ann Surg.

[18] Biere SS, Maas KW, Bonavina L, Garcia JR, van Berge Henegouwen MI, Rosman C, Sosef MN, de Lange ESM, Bonjer HJ, Cuesta MA, van der Peet DL (2011). Traditional invasive vs. minimally invasive esophagectomy: a multi-center, randomized trial (TIME-trial). BMC Surg.

[19] Straatman J, van der Wielen N, Cuesta MA, Daams F, Roig Garcia J, Bonavina L, Rosman C, van Berge Henegouwen MI, Gisbertz SS, van der Peet DL (2017). Minimally invasive versus open esophageal resection: three-year follow-up of the previously reported randomized controlled trial: the TIME trial. Ann Surg.

[20] Biere SS, van Berge Henegouwen MI, Maas KW, Bonavina L, Rosman C, Garcia JR, Gisbertz SS, Klinkenbijl JHG, Hollmann MW, de Lange ESM, Bonjer HJ, van der Peet DL, Cuesta MA (2012). Minimally invasive versus open oesophagectomy for patients with oesophageal cancer: a multicentre, open-label, randomised controlled trial. Lancet.

[21] Burdall OC, Boddy AP, Fullick J, Blazeby J, Krysztopik R, Streets C, Hollowood A, Barham CP, Titcomb D (2015). A comparative study of survival after minimally invasive and open oesophagectomy. Surg Endosc.

[22] Yibulayin W, Abulizi S, Lv H, Sun W (2016). Minimally invasive oesophagectomy versus open esophagectomy for resectable esophageal cancer: a meta-analysis. World J Surg Oncol.

[23] Dantoc M, Cox MR, Eslick GD (2012). Evidence to support the use of minimally invasive esophagectomy for esophageal cancer: a meta-analysis. Arch Surg.

[24] Guo W, Ma X, Yang S, Zhu X, Qin W, Xiang J, Lerut T, Li H (2016). Combined thoracoscopic-laparoscopic esophagectomy versus open esophagectomy: a meta-analysis of outcomes. Surg Endosc.

[25] Xiong WL, Li R, Lei HK, Jiang ZY (2017). Comparison of outcomes between minimally invasive oesophagectomy and open oesophagectomy for oesophageal cancer. ANZ J Surg.

[26] Lv L, Hu W, Ren Y, Wei X (2016). Minimally invasive esophagectomy versus open esophagectomy for esophageal cancer: a meta-analysis. Onco Targets Ther.

[27] Sgourakis G, Gockel I, Radtke A, Musholt TJ, Timm S, Rink A, Tsiamis A, Karaliotas C, Lang H (2010). Minimally invasive versus open esophagectomy: meta-analysis of outcomes. Dig Dis Sci.

[28] Nagpal K, Ahmed K, Vats A, Yakoub D, James D, Ashrafian H, Darzi A, Moorthy K, Athanasiou T (2010). Is minimally invasive surgery beneficial in the management of esophageal cancer? A meta-analysis. Surg Endosc.

[29] van Workum F, Stenstra MHBC, Berkelmans GHK, Slaman AE, van Berge Henegouwen MI, Gisbertz SS, van den Wildenberg FJH, Polat F, Irino T, Nilsson M, Nieuwenhuijzen GAP, Luyer MD, Adang EM, Hannink G, Rovers MM, Rosman C (2019). Learning curve and associated morbidity of minimally invasive esophagectomy: a retrospective multicenter study. Ann Surg.

[30] Sihag S, Kosinski AS, Gaissert HA, Wright CD, Schipper PH (2016). Minimally invasive versus open esophagectomy for esophageal cancer: a comparison of early surgical outcomes from the Society of Thoracic Surgeons National Database. Ann Thorac Surg.

[31] Seesing MFJ, Gisbertz SS, Goense L, van Hillegersberg R, Kroon HM, Lagarde SM, Ruurda JP, Slaman AE, van Berge Henegouwen MI, Wijnhoven BPL (2017). A propensity score matched analysis of open versus minimally invasive transthoracic esophagectomy in the Netherlands. Ann Surg.

[32] Mamidanna R, Bottle A, Aylin P, Faiz O, Hanna GB (2012). Short-term outcomes following open versus minimally invasive esophagectomy for cancer in England: a population-based national study. Ann Surg.

[33] Takeuchi H, Miyata H, Ozawa S, Udagawa H, Osugi H, Matsubara H, Konno H, Seto Y, Kitagawa Y (2017). Comparison of short-term outcomes between open and minimally invasive esophagectomy for esophageal cancer using a nationwide database in Japan. Ann Surg Oncol.

[34] Avery KN, Metcalfe C, Berrisford R, Barham C, Donovan JL, Elliott J, Falk SJ, Goldin R, Hanna G, Hollowood AA, Krysztopik R, Noble S, Sanders G, Streets CG, Titcomb DR, Wheatley T, Blazeby JM (2014). The feasibility of a randomized controlled trial of esophagectomy for esophageal cancer--the ROMIO (Randomized Oesophagectomy: Minimally Invasive or Open) study: protocol for a randomized controlled trial. Trials.

[35] Metcalfe C, Avery K, Berrisford R, Barham P, Noble SM, Fernandez AM, Hanna G, Goldin R, Elliott J, Wheatley T, Sanders G, Hollowood A, Falk S, Titcomb D, Streets C, Donovan JL, Blazeby JM (2016). Comparing open and minimally invasive surgical procedures for oesophagectomy in the treatment of cancer: the ROMIO (Randomised Oesophagectomy: Minimally Invasive or Open) feasibility study and pilot trial. Health Technol Assess.

[36] van Hillegersberg R, Boone J, Draaisma WA, Broeders IAMJ, Giezeman MJMM, Rinkes IHMB (2006). First experience with robot-assisted thoracoscopic esophagolymphadenectomy for esophageal cancer. Surg Endosc.

[37] Kernstine KH, DeArmond DT, Karimi M, van Natta TL, Campos JC, Yoder MR, Everett JE (2004). The robotic, 2-stage, 3-field esophagolymphadenectomy. J Thorac Cardiovasc Surg.

[38] Boone J, Schipper MEI, Moojen WA, Borel Rinkes IHM, Cromheecke GJE, van Hillegersberg R (2009). Robot-assisted thoracoscopic oesophagectomy for cancer. Br J Surg.

[39] van der Sluis PC, Ruurda JP, van der Horst S, Verhage RJJ, Besselink MGH, Prins MJD, Haverkamp L, Schippers C, Rinkes IHMB, Joore HCA, ten Kate FJW, Koffijberg H, Kroese CC, van Leeuwen MS, Lolkema MPJK, Reerink O, Schipper MEI, Steenhagen E, Vleggaar FP, Voest EE, Siersema PD, van Hillegersberg R (2012). Robot-assisted minimally invasive thoraco-laparoscopic esophagectomy versus open transthoracic esophagectomy for resectable esophageal cancer, a randomized controlled trial (ROBOT trial). Trials.

[40] van der Sluis PC, van der Horst S, May AM, Schippers C, Brosens LAA, Joore HCA, Kroese CC, Haj Mohammad N, Mook S, Vleggaar FP, Borel Rinkes IHM, Ruurda JP, van Hillegersberg R (2019). Robot-assisted minimally invasive thoracolaparoscopic esophagectomy versus open transthoracic esophagectomy for resectable esophageal cancer: a randomized controlled trial. Ann Surg.

[41] Sarkaria IS, Rizk NP, Goldman DA, Sima C, Tan KS, Bains MS, Adusumilli PS, Molena D, Bott M, Atkinson T, Jones DR, Rusch VW (2019). Early quality of life outcomes after robotic-assisted minimally invasive and open esophagectomy. Ann Thorac Surg.

[42] Grimminger P. et al. RAMIE Versus MIE for Resectable Esophageal Cancer, a Randomized Controlled Trial (ROBOT-2 Trial). (ROBOT-2) ClinicalTrials.gov Identifier: NCT04306458

[43] Chao YK, Li ZG, Wen YW, Kim DJ, Park SY, Chang YL, van der Sluis PC, Ruurda JP, van Hillegersberg R (2019). Robotic-assisted esophagectomy vs video-assisted thoracoscopic esophagectomy (REVATE): study protocol for a randomized controlled trial. Trials.

[44] Zhang Y, Han Y, Gan Q, Xiang J, Jin R, Chen K, Che J, Hang J, Li H (2019). Early outcomes of robot-assisted versus thoracoscopic-assisted Ivor Lewis esophagectomy for esophageal cancer: a propensity score-matched study. Ann Surg Oncol.

[45] Grimminger PP, Hadzijusufovic E, Lang H (2018). Robotic-assisted Ivor Lewis esophagectomy (RAMIE) with a standardized intrathoracic circular end-to-side stapled anastomosis and a team of two (surgeon and assistant only). Thorac Cardiovasc Surg.

[46] Grimminger PP, Hadzijusufovic E, Ruurda JP, Lang H, van Hillegersberg R (2018). The da Vinci Xi robotic four-arm approach for robotic-assisted minimally invasive esophagectomy. Thorac Cardiovasc Surg.

[47] Grimminger PP, Lang H (2018). Totally minimally invasive esophagectomy and gastric pull-up reconstruction with an intrathoracic circular stapled anastomosis with a team of two (surgeon and assistant only). Thorac Cardiovasc Surg.

[48] Deng J, Su Q, Ren Z, Wen J, Xue Z, Zhang L, Chu X (2018). Comparison of short-term outcomes between minimally invasive McKeown and Ivor Lewis esophagectomy for esophageal or junctional cancer: a systematic review and meta-analysis. Onco Targets Ther.

[49] Zhou C (2015). Is minimally invasive esophagectomy effective for preventing anastomotic leakages after esophagectomy for cancer? A systematic review and meta-analysis. World J Surg Oncol.

[50] Grimminger PP, Goense L, Gockel I, Bergeat D, Bertheuil N, Chandramohan SM, Chen KN, Chon SH, Denis C, Goh KL, Gronnier C, Liu JF, Meunier B, Nafteux P, Pirchi ED, Schiesser M, Thieme R, Wu A, Wu PC, Buttar N, Chang AC (2018). Diagnosis, assessment, and management of surgical complications following esophagectomy. Ann N Y Acad Sci.

[51] Schmidt HM, Gisbertz SS, Moons J, Rouvelas I, Kauppi J, Brown A, Asti E, Luyer M, Lagarde SM, Berlth F, Philippron A, Bruns C, Hölscher A, Schneider PM, Raptis DA, Henegouwen MIB, Nafteux P, Nilsson M, Räsanen J, Palazzo F, Rosato E, Mercer S, Bonavina L, Nieuwenhuijzen G, Wijnhoven BPL, Schröder W, Pattyn P, Grimminger PP, Gutschow CA (2017). Defining benchmarks for transthoracic esophagectomy: a multicenter analysis of total minimally invasive esophagectomy in low risk patients. Ann Surg.

[52] van der Sluis PC, Tagkalos E, Hadzijusufovic E, Babic B, Uzun E, van Hillegersberg R, Lang H, Grimminger PP. (2020) Robot-assisted minimally invasive esophagectomy with intrathoracic anastomosis (Ivor Lewis): promising results in 100 consecutive patients (the European experience). J Gastrointest Surg. 10.1007/s11605-019-04510-8 10.1007/s11605-019-04510-8 PMC7850999 32072382

[53] van der Sluis PC, Ruurda JP, van der Horst S, Goense L, van Hillegersberg R (2018). Learning curve for robot-assisted minimally invasive thoracoscopic esophagectomy: results from 312 cases. Ann Thorac Surg.

[54] van Heijl M, Gooszen JA, Fockens P, Busch OR, Jan van Lanschot J, van Berge Henegouwen MI (2010). Risk factors for development of benign cervical strictures after esophagectomy. Ann Surg.

[55] Markar SR, Karthikesalingam A, Vyas S, Hashemi M, Winslet M (2011). Hand-sewn versus stapled oesophago-gastric anastomosis: systematic review and meta-analysis. J Gastrointest Surg.

[56] Liu QX, Min JX, Deng XF, Dai JG (2014). Is hand sewing comparable with stapling for anastomotic leakage after esophagectomy? A meta-analysis. World J Gastroenterol.

[57] Zhou D, Liu QX, Deng XF, Min JX, Dai JG (2015). Comparison of two different mechanical esophagogastric anastomosis in esophageal cancer patients: a meta-analysis. J Cardiothorac Surg.

[58] Parry K, Haverkamp L, Bruijnen RCG, Siersema PD, Ruurda JP, van Hillegersberg R (2015). Surgical treatment of adenocarcinomas of the gastro-esophageal junction. Ann Surg Oncol.

[59] Visser E, Rossum PSN, Ruurda JP, Hillegersberg R (2017). Impact of lymph node yield on overall survival in patients treated with neoadjuvant chemoradiotherapy followed by esophagectomy for cancer: a population-based cohort study in the Netherlands. Ann Surg.

[60] Visser E, Markar SR, Ruurda JP, Hanna GB, van Hillegersberg R (2019). Prognostic value of lymph node yield on overall survival in esophageal cancer patients: a systematic review and meta-analysis. Ann Surg.

[61] Tagkalos E, Goense L, Hoppe-Lotichius M, Ruurda JP, Babic B, Hadzijusufovic E, Kneist W, van der Sluis PC, Lang H, van Hillegersberg R, Grimminger PP (2020) Robot-assisted minimally invasive esophagectomy (RAMIE) compared to conventional minimally invasive esophagectomy (MIE) for esophageal cancer: a propensity-matched analysis. Dis Esophagus 33(4):doz060. 10.1093/dote/doz060 10.1093/dote/doz060 31206577

[62] Kunzli HT (2017). Pilot-study on the feasibility of sentinel node navigation surgery in combination with thoracolaparoscopic lymphadenectomy without esophagectomy in early esophageal adenocarcinoma patients. Dis Esophagus.

[63] van Hillegersberg R, Seesing MF, Brenkman HJ, Ruurda JP (2017). Robot-assisted minimally invasive esophagectomy. Chirurg.

